# Associations of triglyceride levels with longevity and frailty: A Mendelian randomization analysis

**DOI:** 10.1038/srep41579

**Published:** 2017-01-30

**Authors:** Zuyun Liu, Stephen Burgess, Zhengdong Wang, Wan Deng, Xuefeng Chu, Jian Cai, Yinsheng Zhu, Jianming Shi, Xuejuan Xie, Yong Wang, Li Jin, Xiaofeng Wang

**Affiliations:** 1 Unit of epidemiology, State Key Laboratory of Genetic Engineering and Ministry of Education Key Laboratory of Contemporary Anthropology, School of Life Sciences, Fudan University, 200433, Shanghai, China; 2 Department of Public Health and Primary Care, University of Cambridge, Cambridge, UK; 3 Rugao People’s Hospital, Rugao 226500 Jiangsu, China; 4 Department of Neurology, First Affiliated Hospital, Xinjiang Medical University, Urumqi, 830011, China

## Abstract

Observational studies suggest associations of triglyceride levels with longevity and frailty. This study aimed to test whether the associations are causal. We used data from the Rugao Longevity and Ageing Study, a population-based cohort study performed in Rugao, China. A variant in the *APOA5* gene region (rs662799) was used as the genetic instrument. Mendelian randomization (MR) analyses were performed to examine the associations of genetically predicted triglycerides with two ageing phenotypes – longevity ( ≥95 years) and frailty (modified Fried frailty phenotype and Rockwood frailty index). C allele of rs662799 was robustly associated with higher triglyceride levels in the comparison group (β = 0.301 mmol/L per allele, p < 0.001), with an F statistic of 95.3 and R^2^ = 0.040. However MR analysis did not provide strong evidence for an association between genetically predicted triglyceride levels and probability of longevity (OR: 0.61; 95% CI: 0.35, 1.07 per 1 mmol/L increase in triglycerides). In the ageing arm (70–84 years), genetically predicted triglyceride levels were not associated with the frailty index (β = 0.008; 95% CI: −0.013, 0.029) or the frailty phenotype (OR: 1.91; 95% CI: 0.84, 4.37). In conclusion, there is currently a lack of sufficient evidence to support causal associations of triglyceride levels with longevity and frailty in elderly populations.

Longevity implies exceptional survival and has been considered as a rare but important aging phenotype^1^. Exploiting long-lived individuals (LLIs) (e.g., aged 95+years) as models is well established in human ageing studies^2^. On the other hand, frailty signifies a state of increased vulnerability to minor stressor events due to cumulative declines in many pathophysiological systems during ageing and increases in risks of adverse health outcomes, such as mortality^3^,^4^. To some extent, frailty and longevity represent two complimentary aspects in ageing research studies. Investigations into aetiological factors (e.g., blood biomarkers) that contribute to longevity and frailty would help to elucidate potential pathophysiological mechanisms of ageing.

Recently, observational studies have suggested associations of triglyceride levels with longevity and frailty^5^,^6^. However, common limitations such as potential confounding or reverse causation have made it difficult for observational studies to identify the causal roles of biomarkers in the pathological process of an outcome^7^. In this context, based on the random assignment of genotype from parents that occurs before conception, the Mendelian randomization (MR) approach has been proposed to strengthen causal inferences using genetic variants associated with an exposure (e.g., biomarker) as instrumental variables (e.g., unrelated to potential confounding factors)^7^. The principle has been previously applied to triglycerides, which was confirmed as a causal risk factor for coronary heart disease (CHD)^8^. In that study, one variant in the *APOA5* gene region (−1131 T > C, rs662799) was used as a proxy of triglyceride concentration^8^. Indeed, *APOA5* rs662799 is in almost complete linkage disequilibrium with two other *APOA5* polymorphisms: rs2266788 ( +1891 T > C) and rs651821 (−3 A > G); the haplotype constructed by them was found to be associated with approximately 50% lower *APOA5* gene expression^8^. In view of the adverse effect of cardiovascular diseases such as CHD on ageing, we examined the causal role of triglyceride level in longevity and frailty using the MR approach. In this study, we aimed to provide evidence for understanding the pathogenesis of ageing and thus potential mechanisms for targeted intervention.

Based on data from the Rugao Longevity and Ageing Study (RuLAS), we used MR analysis to determine whether the observational associations of triglyceride levels with the two ageing phenotypes, longevity and frailty, are causal. The aforementioned genetic variant rs662799 was used as the genetic instrument.

## Results

Table 1 presents the socio-demographic characteristics of LLIs and the comparison group. Relative to comparison group, LLIs were more likely to be illiterate (82.0% vs. 51.2%) and to be regular drinkers (38.5% vs. 29.8%) but were less likely to be regular smokers (19.6% vs. 26.6%). The mean values of body mass index (21.5 kg/m^2^ vs. 24.1 kg/m^2^), HDL-C (1.38 mmol/L vs. 1.45 mmol/L), LDL-C (2.51 mmol/L vs. 2.82 mmol/L) and triglyceride levels (1.09 mmol/L vs. 1.43 mmol/L) were lower for LLIs.

### Analysis of longevity as outcome in case-control design

The *APOA5* variant was distributed consistently with Hardy–Weinberg equilibrium (p = 0.744) in the comparison group (Table 2). Frequencies of variant rs662799 were 70.9% (T allele) and 29.1% (C allele). C allele of rs662799 was robustly associated with higher triglyceride levels (beta coefficient (β) = 0.301 mmol/L per allele, p < 0.001), with an F statistic of 95.3 and R^2^ = 0.040. As shown in Table 3, the *APOA5* variant was not associated with the potential confounders listed here.

Each additional copy of the C allele of this variant rs662799 was not strongly associated with probability of longevity (Odds ratio (OR): 0.86; 95% confidence interval (CI): 0.73, 1.02, Table 4). MR analysis revealed that genetically predicted triglycerides were also not associated with probability of longevity (OR: 0.61; 95% CI: 0.35, 1.07 per 1 mmol/L increase in triglycerides). The associations above did not change after further adjustments for potential confounders and bootstrapping with 1000 replications (Table 4). For comparison, serum triglycerides were strongly observationally associated with longevity (OR: 0.52; 95% CI: 0.43, 0.62, p < 0.001). The association remained after further adjustments for confounders.

### Analysis of frailty as outcome in cross-sectional design

The socio-demographic characteristics of the study participants from the ageing arm stratified by *APOA5* genotype are shown in Table S1 (Supplementary Data). The *APOA5* variant was consistent with Hardy–Weinberg equilibrium (p = 0.869). Frequencies of the variant rs662799 in the study sample were 71.9% (T allele) and 28.1% (C allele). The *APOA5* variant was not associated with the potential confounders.

Triglycerides were found to be significantly observationally associated with frailty index (FI) (β = 0.007, p = 0.001) based on a cross-sectional design in the ageing arm of the RuLAS; however, a non-significant observational association was found for the frailty phenotype (OR: 0.99; 95% CI: 0.84, 1.16, p = 0.856). Thus, we used standard two-stage regression analysis to test the association of genetically predicted triglycerides on FI in participants from the ageing arm. These results are presented in Table 5. Genetically predicted triglycerides were not associated with FI (β = 0.008; 95% CI: −0.013, 0.029, p = 0.440) in an unadjusted model. We also did not observe any association of rs662799 with FI (β = 0.003; 95% CI: −0.004, 0.009, p = 0.442). Further adjustments for potential confounders and bootstrapping with 1000 replications did not change the non-significant associations (Table 5). A MR analysis revealed that genetically predicted triglycerides were also not strongly associated with the frailty phenotype (OR: 1.91; 95% CI: 0.84, 4.37, p = 0.124) (Table S2, model 1). The genetic instrument rs662799 was not associated with frailty phenotype (OR: 1.20; 95% CI: 0.95, 1.52, p = 0.124) (Table S2, model 1). The associations did not change after further adjustments for potential confounders and bootstrapping with 1000 replications (Table S2).

We also performed sensitivity analysis by combining the elderly group in the longevity arm and elderly participants in the ageing arm as controls for longevity. Similar results were observed, including a strong association of the rs662799 variant with triglyceride levels and absence of evidence for a causal association of triglycerides with longevity.

## Discussion

The specific study design in RuLAS provides us with the unique ability to simultaneously examine determinants of longevity and ageing^9^,^10^. This study used MR analysis to examine the causal associations of triglyceride levels with two ageing phenotypes (longevity and frailty). We observed that the genetic instrument rs662799 was robustly associated with triglyceride levels. Moreover, triglyceride levels were also associated with longevity and frailty (in particular, FI). However, rs662799 and genetically elevated triglycerides were not associated with the probability of longevity nor with frailty. The present results do not support causal associations of triglyceride levels with longevity and frailty.

In this study, triglycerides were found to be related to longevity, which is consistent with previous studies using long-lived persons^11^ or their offspring^6^ as models of longevity. For instance, Vaarhorst *et al*., observed that offspring of nonagenarian siblings from 421 families of the Leiden Longevity Study had lower triglycerides levels than their partners^6^. For frailty, we focused on two of several definitions that have been widely used, including the cumulative deficit model and phenotype model^3^,^12^,^13^,^14^. In this study, only FI was found to be associated with triglyceride levels. With one exception^5^, no prior study has observed the presence of the association between frailty phenotype and triglyceride levels^15^,^16^,^17^,^18^. Note that this only study targeted on 1622 British males aged 71–92 years without established cardiovascular disease^5^. The discrepancy between the triglyceride associations with FI and frailty phenotype might be attributed to differences in characteristics of the study populations (e.g., ethnics, sample size) and the criteria for the definition of frailty. For example, it is reported that FI captures the broader spectrum of disorders and could better identify elderly adults at high risk for adverse outcomes than the frailty phenotype does in the early stages of frailty^3^. Discussion on the two frailty measurements is beyond the current study but further comparisons and distinctions are needed to elucidate this issue.

Our results suggest that the observational associations of triglyceride levels with longevity and frailty were not causal. This result appears to be reasonable when taking into account studies on genetic biomarkers of longevity^19^,^20^,^21^,^22^, FI and frailty phenotype^23^,^24^. These findings indicate that the observational associations of triglycerides with longevity and FI might involve other factors, e.g., cardiovascular disease. First, triglyceride is synthesized in intestinal and liver cells and is subsequently transported through the plasma and acts as a key energy source^25^. Increased triglyceride levels may be related to increased levels of remnant lipoproteins, which may initiate and promote the development of atherosclerotic plaques^26^. These findings could explain the fact that elevated triglyceride levels have been considered as a major independent risk factor for cardiovascular disease^8^,^26^,^27^, a leading cause of death in the elderly^28^,^29^,^30^,^31^. Second, it has been proposed that LLIs with extended life span have escaped, delayed, or survived major age-related diseases, such as cardiovascular disease^32^. The above assumption is intriguing and evidently, requires further scrutiny.

The MR method has some inherent qualifications. For instance, in this study, consistent with other studies^33^, the rs662799 was strongly associated with triglyceride levels in the comparison group but was not associated with the potential confounders listed here, indicating that the genetic instrumental variable satisfies the first two assumptions in the MR analysis^7^. Regression analysis in the first stage obtained an F statistic of 95.3 and an R^2^ of 0.040 (that is, the genetic variant explains approximately 4% of the between individual variance in triglyceride levels) and confirmed that the variant rs662799 could be considered a strong genetic instrument^8^. In addition, the instrument should be related to the outcome (i.e., longevity and frailty) only through its direct effect on modifiable exposure (i.e., triglyceride levels)^7^,^34^. Note that a weak association of the variant rs662799 with HDL-C levels was observed in this study (β = −0.067, Table 2). According to previous research, the non-triglyceride-mediated pathway would be not excluded and the pleiotropy of rs662799 would be existed. This might be a limitation of similar studies, including the current one. However, rs662799 “is a regulatory variant of APOA5 that is predominantly associated with triglyceride concentration”, and the scope for pleiotropy is reduced^8^. Given the limited choice of variants associated with triglyceride concentration, several (unlinked) genetic variants need to be used in further studies.

The RuLAS has a unique strength for simultaneously examining factors associated with longevity and frailty – two important measurable ageing phenotypes. Participants in the two study arms were from the same city and showed a similar genomic background. In addition, the controls were sampled from the original population of the case group, which is a pivotal strength for the case-control design because they encounter homogeneous environmental exposures.

However, some limitations need to be mentioned. First, the sample size is relatively small (although LLIs in this study represent 65.7% of the Rugao LLIs) for MR analysis, potentially leading to biased estimates. Although the between individual variance in triglyceride concentrations explained by the genetic variant in our study is comparable to those of other similar reports^8^, we cannot exclude the possibility that the absence of associations between genetically predicted triglycerides and longevity and frailty is due to the small sample size. However, we observed that no association of the *APOA5* gene or the variant rs662799 with longevity or ageing was reported in studies using the conventional candidate gene approach^19^,^20^ or GWAS^21^,^22^. Second, some potential confounding factors, such as data on diet were not available, and we cannot adjust for these factors at this moment. However, the RuLAS is ongoing, and subsequent re-surveys and follow-ups are anticipatory.

In conclusion, there is currently a lack of sufficient evidence to support causal associations of triglycerides with longevity and frailty in the Rugao elderly population. These findings warrant further validation in other cohorts with a larger sample size.

## Methods

### Study design

The study design included two components (Figure S1). First, based on a case-control design, we examined the associations of triglyceride levels, rs662799, and genetically predicted triglycerides (MR method) with longevity. Second, we tested similar associations for frailty based on a cross-sectional design.

### Study population and procedure

All data used in this study were obtained from the RuLAS, a population-based observational two-arm cohort study performed in Rugao, a typical medium-sized city of Jiangsu province, China. A detailed description was provided in our previous publication^9^. Briefly, the longevity arm recruited 463 long-lived participants (103 men and 360 women, aged 95 +years of age, range 95–107 years) between December 2007 and February 2008, representing 65.7% of the Rugao LLIs. Furthermore, population-based control groups were randomly recruited from the resident registry at the local government offices of Rugao in this arm. The ageing arm of the RuLAS randomly recruited approximately 1960 elderly participants aged 70‒84 years from 31 rural communities of Jiang’an Township, Rugao city, between November 2014 and December 2014 according to 5-year age and sex strata. Finally, the sample consisted of a total of 1788 participants (91.2%). The current study used data on long-lived participants and the control elderly group in the longevity arm and 1750 elderly participants in the ageing arm after data cleaning and a full consideration of study designs.

As previously described^9^,^35^, a detailed structured questionnaire and physical examinations (there was a slight difference in the two arms) were administered by trained physicians and information such as socio-demographic characteristics, lifestyles, past medical history, psychological health, common health deficits (e.g. urinary incontinence), social support/relations, cognitive function, and depression, etc. were gathered^9^. For parts of older adults with cognitive impairment, an appropriate proxy was asked to help accomplish the questionnaire. Written informed consent was obtained from each participant or a member of his/her immediate family. The research was approved by the Human Ethnics Committee of Fudan University School of Life Sciences and was performed in accordance with the approved guidelines.

In this study, the following information was obtained: age, sex, marital status, education level, lifestyles (smoking and drinking habits), body mass index, and blood pressure. Marital status was categorized as currently married or other. Education level was categorized as illiterate or literate (≥1 year of education). A participant was categorized as a regular smoker (ever) if he/she reported “Yes” to the question, “Have you ever smoked continuously for more than 6 months ? ” or as non-smoker if he/she reported “No.” The same criteria were used to define drinking habits. Body mass index was calculated as weight divided by height squared (kg/m^2^).

### Genetic instrument

In East Asians, several SNPs in the *APOA5* gene associated with triglyceride levels including rs662799, rs651821 (−3 A > G), rs3135506 (S19W, c.56 C > G), rs2072560 (715 G > T, IVS3 + 476 G > T), rs2266788 (1891 T > C, c.158 C > T, c.1259 T > C), and rs2075291 (c.553 G > T, G185C); while four of them (rs662799, rs651821, rs2072560, and rs2266788) were in strong LD^36^, and the SNP rs662799, a regulatory variant in the promoter region of the *APOA5*, can be the representative of the other three SNPs. Additionally, every C allele inherited of the SNP rs662799 was associated with mean triglyceride concentration of 16%^8^. Therefore, the variant −1131 T > C (rs662799) was used as the genetic instrument in this study.

### Triglyceride levels

One aliquot of serum was used for clinical testing by a technician in the biochemistry laboratory of Rugao Hospital of Traditional Chinese Medicine (in the longevity arm) or Rugao People’s Hospital (in the ageing arm). In addition to triglycerides, other blood biomarkers such as high-density-lipoprotein cholesterol (HDL-C), and low-density-lipoprotein cholesterol (LDL-C) were also examined.

### Longevity and frailty

For the case-control design, the long-lived participants were cases of longevity, whereas the elderly group in the longevity arm and elderly participants aged 70–79 years in the ageing arm were combined as the comparison group.

In the cross-sectional ageing arm, we used two commonly used approaches to operationalise frailty: FI based on the cumulative deficit model^13^,^14^ and frailty phenotype according to Fried *et al*.^12^. A detailed description of FI construction was provided in our previous publication^10^. Briefly, 45 health deficits, including symptoms, activities of daily living (basic and instrumental), comorbidity, cognitive and psychological function were used to construct a FI according to the standard procedure developed by Searle and Rockwood^13^,^14^. Each deficit was dichotomized or polychotomized and mapped to the interval 0–1 to represent the severity of the deficit. The FI was calculated by summing all deficits and dividing by the total number of deficits (n = 45), with a range from 0 to 1. Continuous FI was used in this study.

According to Fried *et al*.^12^, five components, including unintentional weight loss, weakness, exhaustion, slowness, and low activity were used to define the frailty phenotype. Similar measurements of the five criteria were adopted in our study. Detailed descriptions had been provided in our previous publication^37^. Unintentional weight loss, exhaustion, and low activity were based on self-reports, including “weight has decreased by 4.5 kg or 5% during the last 12 months,” “feeling tired all of the time (at least 3 or 4 days per a week),” and “needing help to walk.” Weakness was based on the self-report of “having difficulty in lifting or carrying something as heavy as 10 kg,” which was similar to that used in other studies^38^. Slowness was defined as being below the 20^th^ sex-specific percentile in gait speed (assessed through a timed ‘up and go’ test). Participants with three or more of the above five components were defined as “frail” or otherwise “non-frail.” Thus, a binary frailty phenotype was used in this study.

### Statistical analyses

Characteristics were expressed as the mean ± standard deviation (SD) or the percentage. The chi-squared was used for comparisons of categorical variables, and either the Mann–Whitney or the Kruskal–Wallis test was used for comparisons of continuous variables. A deviation from Hardy–Weinberg equilibrium for the variant rs662799 was tested using a chi-squared test.

We performed an MR analysis (two-stage regression) to examine the causal association of triglyceride levels with longevity. In stage 1, the association of the variant rs662799 with triglyceride levels was analysed using linear regression analysis in the comparison group and the β-coefficient was documented. The F-statistic from the linear regression analysis of triglycerides on this variant was obtained; F-statistic > 10 suggests that potential bias due to weak instruments should not be substantial^39^. In stage 2, the association of genetically predicted triglycerides (calculated according to the equation from stage 1 in the comparison group) with longevity was analysed using logistic regression (LLIs vs. the comparison group). Four models were performed in stage 2. Model 1 and Model 2were unadjusted and adjusted for sex (age was added for frailty analysis), respectively. Model 3 additionally adjusted for education level, marital status, smoking and drinking habit, body mass index, systolic blood pressure, and diastolic blood pressure. Model 4 used bootstrapping with 1000 replications for internal validation for model 2.

With respect to frailty, the association of the variant rs662799 with triglyceride levels was analysed using linear regression analysis in the ageing arm of RuLAS in stage 1. In stage 2, the association of genetically predicted triglycerides (calculated according to the equation from stage 1 in the ageing arm) with FI was analysed using linear regression analysis, whereas the association with frailty phenotype was tested using logistic regression analysis. Four models similar to that used in longevity analysis were used in the frailty study.

MR analyses were performed using Stata version 12 (Stata Corp, College Station, Texas, USA). Other analyses were performed using SAS software (version 9.3; SAS Institute, Cary, NC). All P values were two-sided, a P value < 0.05 was considered statistically significant except where indicated.

## Additional Information

**How to cite this article**: Liu, Z. *et al*. Associations of triglyceride levels with longevity and frailty: A Mendelian randomization analysis. *Sci. Rep.* **7**, 41579; doi: 10.1038/srep41579 (2017).

**Publisher's note:** Springer Nature remains neutral with regard to jurisdictional claims in published maps and institutional affiliations.

## Supplementary Material

Supplementary Information

## Figures and Tables

**Table 1:** Socio-demographic characteristics of long-lived individuals and the comparison group.

	No.^†^	Long-lived individuals^†^	Comparison group^†^	P Value^‡^
Age, years, mean (SD)	2732	97.4 (2.1)	70.6 (5.3)	<0.001
Female, n (%)	2732	343 (78.3)	1430 (62.3)	<0.001
Illiterate, n (%)	2707	346 (82.0)	1170 (51.2)	<0.001
Currently married, n (%)	2707	22 (5.2)	1667 (73.0)	<0.001
Regular smoker (ever), n (%)	2731	86 (19.6)	609 (26.6)	0.002
Regular drinker (ever), n (%)	2713	166 (38.5)	681 (29.8)	<0.001
Body mass index, kg/m^2^, mean (SD)	2725	21.5 (4.2)	24.1 (3.4)	<0.001
Systolic blood pressure, mmHg, mean (SD)	2721	136.7 (22.9)	148.1 (25.0)	<0.001
Diastolic blood pressure, mmHg, mean (SD)	2731	80.1 (11.0)	82.0 (13.1)	<0.001
Serum lipids, mmol/L, mean (SD)				
Triglyceride	2721	1.09 (0.50)	1.43 (0.97)	<0.001
HDL-C	2721	1.38 (0.34)	1.45 (0.33)	<0.001
LDL-C	2721	2.51 (0.70)	2.82 (0.75)	<0.001

SD, standard deviation; HDL-C, high-density-lipoprotein cholesterol; LDL-C, low-density-lipoprotein cholesterol. ^†^The numbers vary because of missing data in variables. For example, overall 2732 participants include 438 long-lived individuals and 2294 elderly individuals (comparison group). ^‡^P values are from the Mann–Whitney test for continuous variables or from the chi-squared test for categorical variables, 2-sided.

**Table 2:** Associations of the *APOA5* polymorphism rs662799 with serum lipids in the comparison group (n = 2294).

	Genotype or allele	No.	P Value for HWE	Triglyceride, mmol/L, mean (SD)	β^†^ (SE)	P Value	HDL-C, mmol/L, mean (SD)	β^†^ (SE)	P Value	LDL-C, mmol/L, mean (SD)	β^†^ (SE)	P Value
−1131T > C (rs662799)	TT	1150 (50.1)	0.744	1.29 (0.82)	Referent		1.48 (0.33)	Referent		2.78 (0.73)	Referent	
	TC	953 (41.5)		1.46 (0.86)	0.169 (0.041)	<0.001	1.44 (0.32)	−0.039 (0.014)	0.007	2.87 (0.77)	0.095 (0.033)	0.004
	CC	191 (8.3)		2.09 (1.70)	0.797 (0.074)	<0.001	1.30 (0.31)	−0.177 (0.026)	<0.001	2.78 (0.79)	0.007 (0.058)	0.908
Additive model	T	3253 (70.9)										
	C	1335 (29.1)			0.301 (0.031)	<0.001		−0.067 (0.011)	<0.001		0.042 (0.024)	0.083

HDL-C, high-density-lipoprotein cholesterol; LDL-C, low-density-lipoprotein cholesterol; SE, standard error; HWE, Hardy-Weinberg equilibrium. ^†^β refers to the average change in serum lipids (unit: mmol/L) with each additional copy of the C allele of this variant rs662799 or using as the “TT” genotype as the reference group.

**Table 3:** Socio-demographic characteristics of long-lived individuals and the comparison group by *APOA5* genotype (n = 2732)^†^.

	TT	TC	CC	P Value^‡^
Female, n (%)	897 (64.4)	726 (65.2)	150 (66.7)	0.778
Illiterate, n (%)	780 (56.6)	618 (55.9)	118 (53.2)	0.633
Currently married, n (%)	833 (60.4)	717 (64.8)	139 (62.6)	0.077
Regular smoker (ever), n (%)	363 (26.1)	275 (24.7)	57 (25.3)	0.729
Regular drinker (ever), n (%)	436 (31.6)	344 (31.1)	67 (29.8)	0.863
Body mass index, kg/m^2^, mean (SD)	23.7 (3.7)	23.7 (3.8)	23.4 (3.4)	0.644
Systolic blood pressure, mmHg, mean (SD)	146.4 (25.4)	146.0 (24.7)	146.4 (24.2)	0.665
Diastolic blood pressure, mmHg, mean (SD)	81.9 (13.5)	81.5 (14.4)	81.6 (10.4)	0.425

SD, standard deviation. ^†^The numbers vary because of missing data in variables. Overall 2732 participants include 438 long-lived indiviudals and 2294 elderly individuals (comparison group). ^‡^P-values are from the Kruskal-Wallis test for continuous variables or from the chi-squared test for categorical variables, 2-sided.

**Table 4:** MR analysis for the association of triglyceride with longevity based on a case-control design (n = 2732)^†^.

Model^※^	rs662799 (Per C allele)			MR analysis^‡^		
	OR	95% CI	P value	Causal OR	95% CI	P value
1	0.86	0.73, 1.02	0.077	0.61	0.35, 1.07	0.083
2	0.86	0.73, 1.01	0.063	0.59	0.34, 1.04	0.068
3	0.83	0.67, 1.04	0.103	0.55	0.28, 1.12	0.099
4	0.85	0.72, 1.02	0.074	0.59	0.34, 1.05	0.074

MR, Mendelian randomization; OR, odds ratio; CI, confidence interval. ^†^The numbers vary because of missing data in variables. Overall 2732 participants include 438 long-lived individuals and 2294 elderly individuals (comparison group). ^※^Model 1 unadjusted model; model 2 adjusted for sex; model 3 additionally adjusted for education level, marital status, smoking and drinking habit, body mass index, systolic blood pressure, and diastolic blood pressure; model 4 used bootstrapping with 1000 replications for internal validation for model 2. ^‡^Genetically predicted triglycerides were calculated according to the equation from stage 1 in the comparison group.

**Table 5:** MR analysis for the association of triglyceride with FI based on a cross-sectional design in the ageing arm (n = 1750)^†^.

Model^※^	rs662799 (Per C allele)			MR analysis		
	β^‡^	95% CI	P Value	β^‡^	95% CI	P Value
1	0.003	−0.004, 0.009	0.442	0.008	−0.013, 0.029	0.440
2	0.002	−0.004, 0.008	0.551	0.006	−0.014, 0.026	0.550
3	0.003	−0.004, 0.009	0.426	0.008	−0.011, 0.027	0.425
4	0.002	−0.005, 0.008	0.559	0.006	−0.016, 0.028	0.579

MR, Mendelian randomization; CI, confidence interval; FI, frailty index. ^†^The numbers vary because of missing data in variables. ^※^Model 1 unadjusted model; model 2 adjusted for age and sex; model 3 additionally adjusted for education level, marital status, smoking and drinking habit, body mass index, systolic blood pressure, and diastolic blood pressure; model 4 used bootstrapping with 1000 replications for internal validation for model 2. ^‡^β refers to the average change in FI (no unit) with each additional copy of C allele of this variant rs662799 or each unit (mmol/L) increase in genetically predicted triglycerides.
