# Corrigendum: Associations of triglyceride levels with longevity and frailty: A Mendelian randomization analysis

**DOI:** 10.1038/srep43981

**Published:** 2017-03-23

**Authors:** Zuyun Liu, Stephen Burgess, Zhengdong Wang, Wan Deng, Xuefeng Chu, Jian Cai, Yinsheng Zhu, Jianming Shi, Xuejuan Xie, Yong Wang, Li Jin, Xiaofeng Wang

Scientific Reports 7: Article number: 4157910.1038/srep41579; published online: 01 30 2017; updated: 03 23 2017

The Acknowledgements section in this Article is incomplete.

“We acknowledge all the people who participated in the Rugao Longevity and Ageing study. We acknowledge the support and help from the Yale Program on Aging (Yale School of Medicine) and the Yale Pepper Older Americans Independence Center (OAIC). This work was supported by the National Natural Science Foundation (grant 81571372, 31171216, and 81260180), the National Basic Research Program (grant 2012CB944600), the International cooperation project of Ministry of science and technology (grant 2014DFA32830), the Science and Technology Program of Jiangsu Province (grant BC2012164), and the National Science & Technology Support Program (grant 2011BAI09B00)”.

should read:

“We acknowledge all the people who participated in the Rugao Longevity and Ageing study. Dr. Liu is the recipient of a James Hudson Brown–Alexander B. Coxe Fellowship from Yale University School of Medicine. We acknowledge the support and help from the Yale Program on Aging (Yale School of Medicine) and the Yale Claude D. Pepper Older Americans Independence Center (P30 AG021342). This work was supported by the National Natural Science Foundation (grant 81571372, 31171216, and 81260180), the National Basic Research Program (grant 2012CB944600), the International cooperation project of Ministry of science and technology (grant 2014DFA32830), the Science and Technology Program of Jiangsu Province (grant BC2012164), and the National Science & Technology Support Program (grant 2011BAI09B00)”.

