# Supplementary material for: Associations of triglyceride levels with longevity and frailty: A Mendelian randomization analysis
Source: Sci Rep. 2017 Jan 30;7:41579. doi: 10.1038/srep41579 (PMC5278549; doi:10.1038/srep41579)

**Associations of triglyceride levels with longevity and frailty: A Mendelian randomization analysis**

Zuyun Liu^1,5^, Stephen Burgess^3^, Zhengdong Wang^2^, Wan Deng^1^, Xuefeng Chu^2^, Jian Cai^4^, Yinsheng Zhu^2^, Jianming Shi^2^, Xuejuan Xie^2^, Yong Wang^2^, Li Jin^1*^, Xiaofeng Wang^1*^

**Table S1. Socio-demographic characteristics by APOA5 genotype based on a cross-sectional design in the ageing arm (n=1750)**^†^**.**

|  | All | TT | TC | CC | P Value ^‡^ |
| --- | --- | --- | --- | --- | --- |
| No. | 1750 | 905 | 705 | 140 | - |
| Age, years, mean (SD) | 75.3 (3.9) | 75.4 (3.9) | 75.3 (3.9) | 75.4 (3.6) | 0.719 |
| Female, n (%) | 934 (53.4) | 475 (52.5) | 380 (53.9) | 79 (56.4) | 0.641 |
| Currently married, n (%) | 1150 (65.7) | 588 (65.0) | 470 (66.7) | 92 (65.7) | 0.777 |
| Illiterate, n (%) | 943 (53.9) | 487 (53.8) | 376 (53.3) | 80 (57.1) | 0.710 |
| Regular smoker (ever), n (%) | 445 (25.4) | 226 (25.0) | 183 (26.0) | 36 (25.7) | 0.901 |
| Regular drinker (ever), n (%) | 514 (29.4) | 256 (28.3) | 217 (30.8) | 41 (29.3) | 0.552 |
| Body mass index, mean (SD) | 24.1 (3.5) | 24.2 (3.4) | 24.1 (3.6) | 23.7 (3.6) | 0.190 |
| Systolic blood pressure, mmHg, mean (SD) | 154.6 (26.0) | 154.7 (26.4) | 154.2 (25.8) | 155.6 (24.2) | 0.639 |
| Diastolic blood pressure, mmHg, mean (SD) | 80.8 (14.9) | 80.8 (15.9) | 80.5 (14.2) | 81.6 (11.5) | 0.755 |

SD, standard deviation. ^†^ The numbers vary because of missing data in variables. ^‡^ P values are from the Kruskal-Wallis test for continuous variables or from the chi-squared test for categorical variables, 2-sided.

**Table S2. MR analysis for the association of triglyceride with frailty phenotype based on a cross-sectional design in the ageing arm^†^.**

| Model ^‡^ | rs662799 (Per C allele) | | | MR analysis | | |
| --- | --- | --- | --- | --- | --- | --- |
|  | OR | 95% CI | P Value | Causal OR | 95% CI | P Value |
| 1 | 1.20 | 0.95, 1.52 | 0.124 | 1.91 | 0.84, 4.37 | 0.124 |
| 2 | 1.19 | 0.94, 1.51 | 0.145 | 1.90 | 0.80, 4.48 | 0.145 |
| 3 | 1.23 | 0.97, 1.56 | 0.086 | 2.02 | 0.91, 4.49 | 0.086 |
| 4 | 1.19 | 0.94, 1.52 | 0.149 | 1.90 | 0.78, 4.61 | 0.158 |

MR, Mendelian randomization; OR, odds ratio; CI, confidence interval. ^†^ The total number is n=1643 due to missing values. ^‡^ Model 1 unadjusted model; model 2 adjusted for age and sex; model 3 additionally adjusted for education level, marital status, smoking and drinking habit, body mass index, systolic blood pressure, and diastolic blood pressure; model 4 used bootstrapping with 1000 replications for internal validation for model 2.


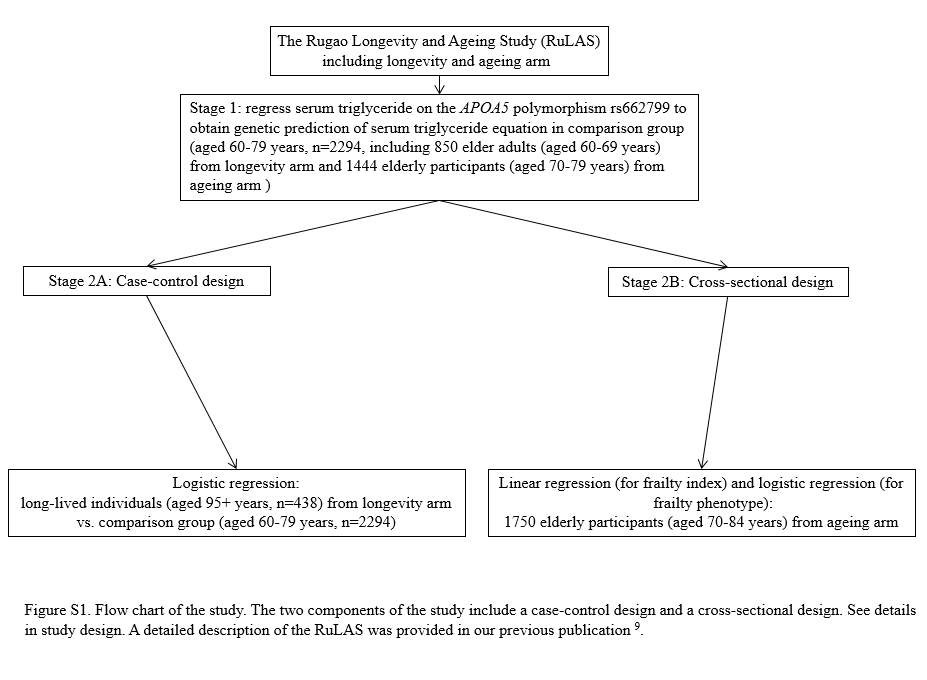

Supplement: Supplementary Information [file srep41579-s1.doc]
